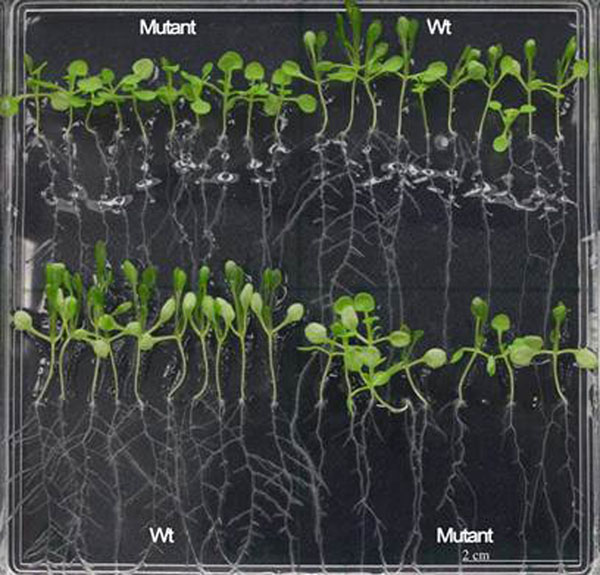# Molecular integration of light responses in *Arabidopsis*

**DOI:** 10.1186/1753-6561-5-S7-P95

**Published:** 2011-09-13

**Authors:** Juan Pablo Matte Risopatron, Yuqiang Sun, Liping Ke, Errin Johnson, Neil Wilson, Amandeep Kour, Steven Swain, Goran Sandberg, Brian Jones

**Affiliations:** 1 Faculty of Agriculture, Food and Natural Resources, University of Sydney, Australia; 2 College of Life and Environmental University, Hangzhou Normal University, China; 3 Plant Industry, CSIRO, Black Mountain, Canberra, Australia; 4 Department of Plant Physiology, Umeå University, Sweden

## 

Growth habit and developmental phase transitions in plants are acutely responsive to the quantity and quality of incident light. Information about neighbouring plants is primarily perceived by changes in the light environment. Arabidopsis is able to distinguish these changes through the phytochrome (PHYA-E) red light receptors that perceive changes in the red to far red (R:FR) ratio, and the cryptochrome and phototropin blue light receptors. Phytochrome, in its biologically active far-red light-absorbing form (Pfr), mediates its effects by directly binding with the basic helix-loop-helix (bHLH) phytochrome-interacting factors (PIFs). High R:FR ratios initiate phytochrome (Pfr)-PIF binding and PIF protein degradation. Low R:FR ratios photo-convert Pfr into the inactive form Pr allowing the gene expression that leads to a range of adaptations, including the shade avoidance responses. PIF1 regulates the expression of a number of genes by directly binding to a G-box element in their promoter. T-DNA insertional mutagenesis of a number of these genes results in pleiotropic effects associated with an altered capacity to respond appropriately to environmental cues, including those signalling changes in soil water status and the ambient light environment. The data show evidence of a perturbation of light regulated response processes, including germination, the shade avoidance responses, stomatal conductance, flowering and the circadian clock perturbation in the PIF-regulated gene mutants. The *pif1,3,4,5* quadruple and one of the PIF-regulated gene mutants fail to show shade avoidance responses in older plants grown under shade inducing conditions. This suggests that the encoded proteins mediate a parallel pathway in the induction of the SAS. The implications of our findings for an understanding of how plants appropriately respond to changes in their environment will be discussed.

**Figure 1:**